# Supplementary material for: STAT3β Enhances Sensitivity to Concurrent Chemoradiotherapy by Inducing Cellular Necroptosis in Esophageal Squamous Cell Carcinoma
Source: Cancers (Basel). 2021 Feb 21;13(4):901. doi: 10.3390/cancers13040901 (PMC7926856; doi:10.3390/cancers13040901)
Supplement: Supplementary file 1 [file cancers-13-00901-s001.pdf]

Supplementary Materials: STAT3β Enhances Sensitivity to Concurrent Chemoradiother-apy by Inducing Cellular Necroptosis in Esophageal Squamous Cell Carcinoma

Zhen-Yuan Zheng, Ping-Lian Yang, Wei Luo, Shuai-Xia Yu, Hong-Yao Xu, Ying Huang, Rong-Yao Li, Yang Chen, Xiu-E Xu, Lian-Di Liao, Shao-Hong Wang, He-Cheng Huang, En-Min Li and Li-Yan Xu

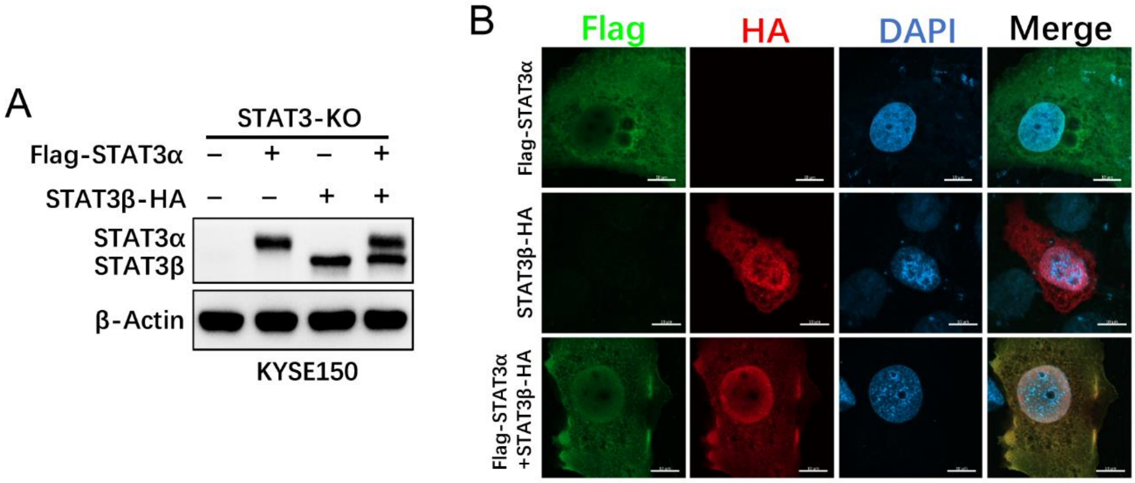

Figure S1. A. KYSE150 STAT3-KO cells transfected with Flag-STAT3α, STAT3β-HA and Flag-STAT3α plus STAT3β-HA, the protein expression were detected by western blotting. B. The location of STAT3α and STAT3β was detected by staining Flag and HA. Scale bar: 10μm. The uncropped Western Blot Figures in Figure S9.

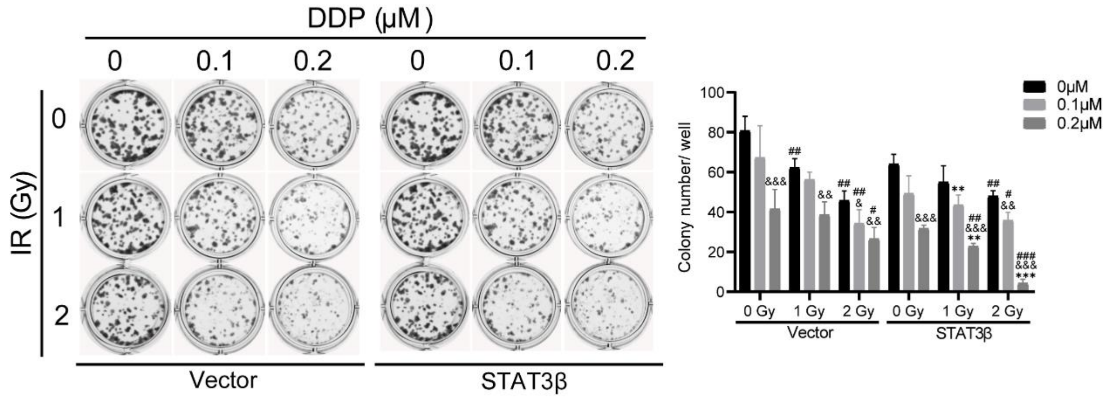

Figure S2. The ability of colony formation of high expressed of empty vector and STAT3β in KYSE510 under cisplatin/ ionizing radiation chemoradiotherapy treatment. Left, the representative diagrams of colony formation. Right, statistical histogram of colony formation number. Error bars represent means ± SEM of three independent experiments. $^{\#}$ $P < 0.05$, $^{\#\#}$ $P < 0.01$, $^{\#\#\#}$ $P < 0.001$ compared with 0 Gy at the same concentration of DDP; $^{\&}$ $P < 0.05$, $^{\&\&}$ $P < 0.01$, $^{\&\&\&}$ $P < 0.01$ compared with 0 μM DDP at the same dose of IR; * $P < 0.05$, ** $P < 0.01$, *** $P < 0.001$ compared with the same Vector treatment.

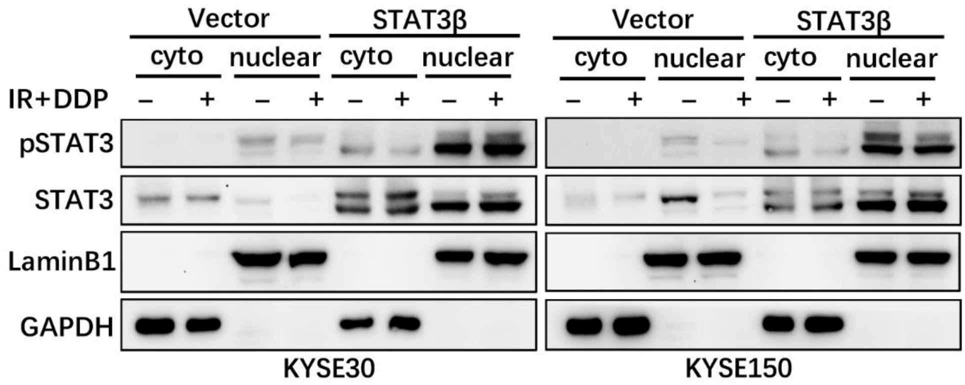

Figure S3. KYSE30 and KYSE150 highly expressing Vector and STAT3β treated with or without cisplatin combined with ionizing radiation, cells were harvested and conducted with nuclear plasma separation. Cyto: cytoplasm. The uncropped Western Blot Figures in Figure S10.

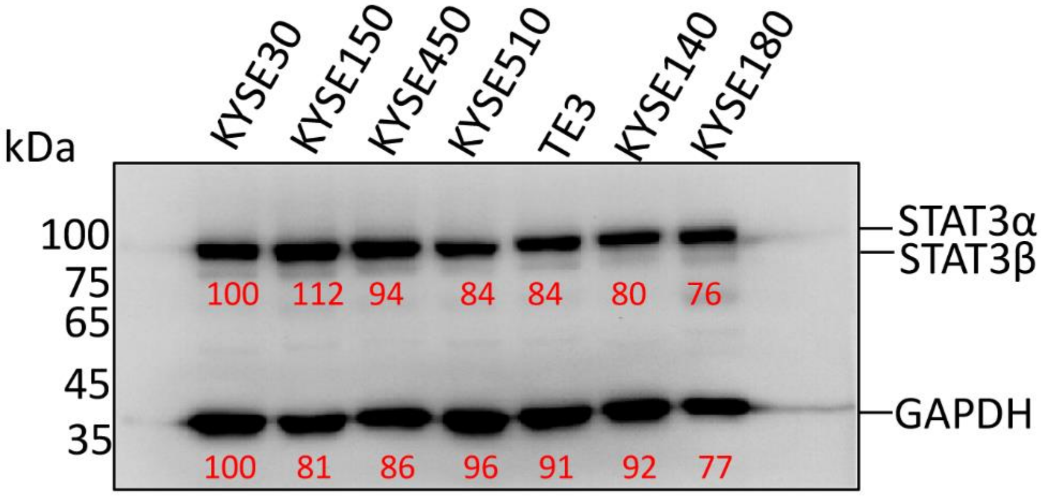

Figure S4. Uncropped Western Blot Images for Figure 3A.

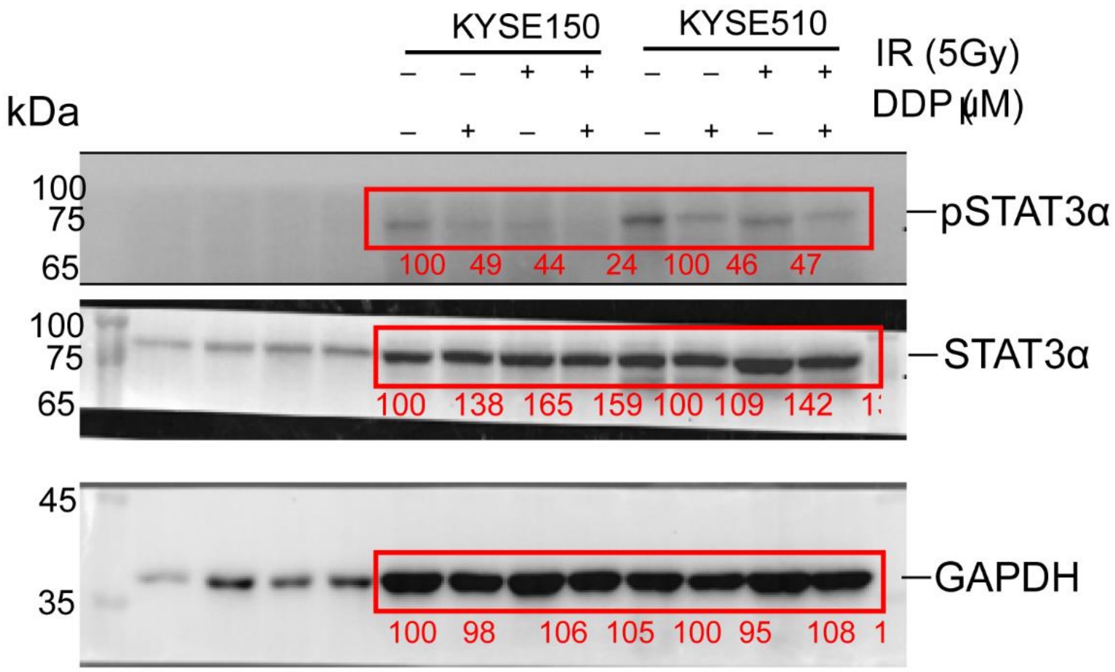

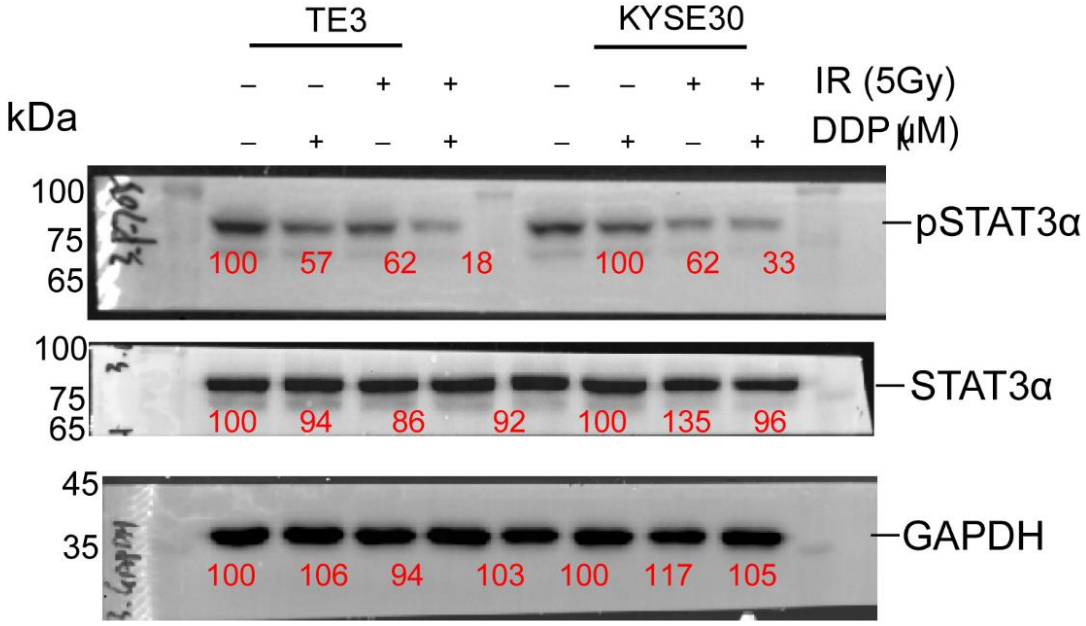

Figure S5. Uncropped Western Blot Images for Figure 3C.

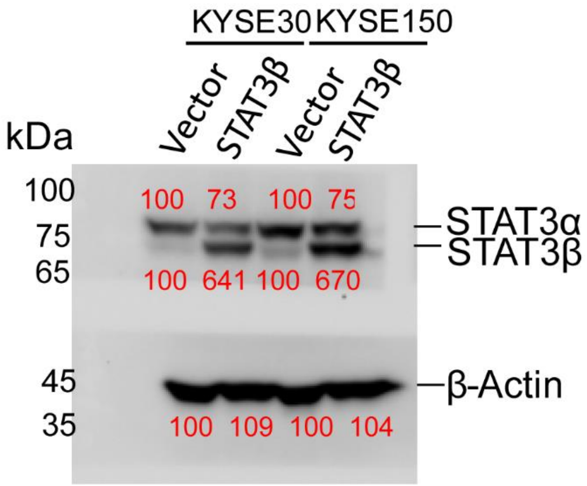

Figure S6. Uncropped Western Blot Images for Figure 4A.

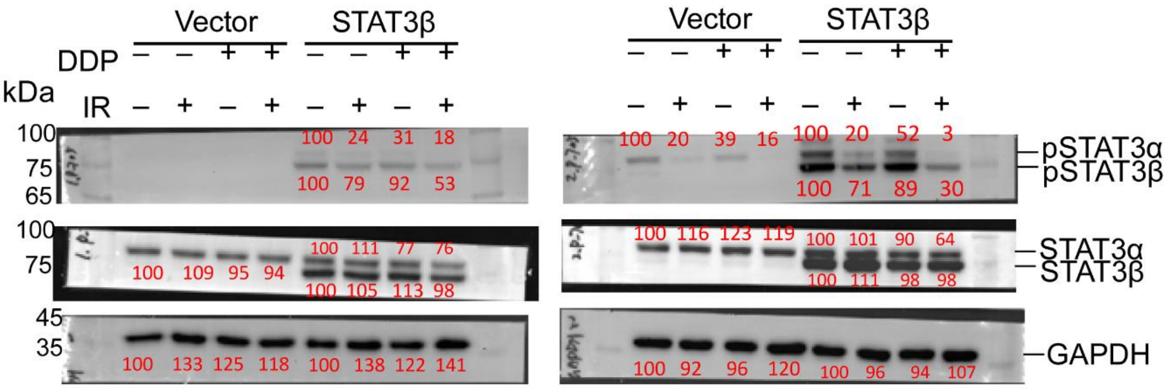

Figure S7. Uncropped Western Blot Images for Figure 4B.

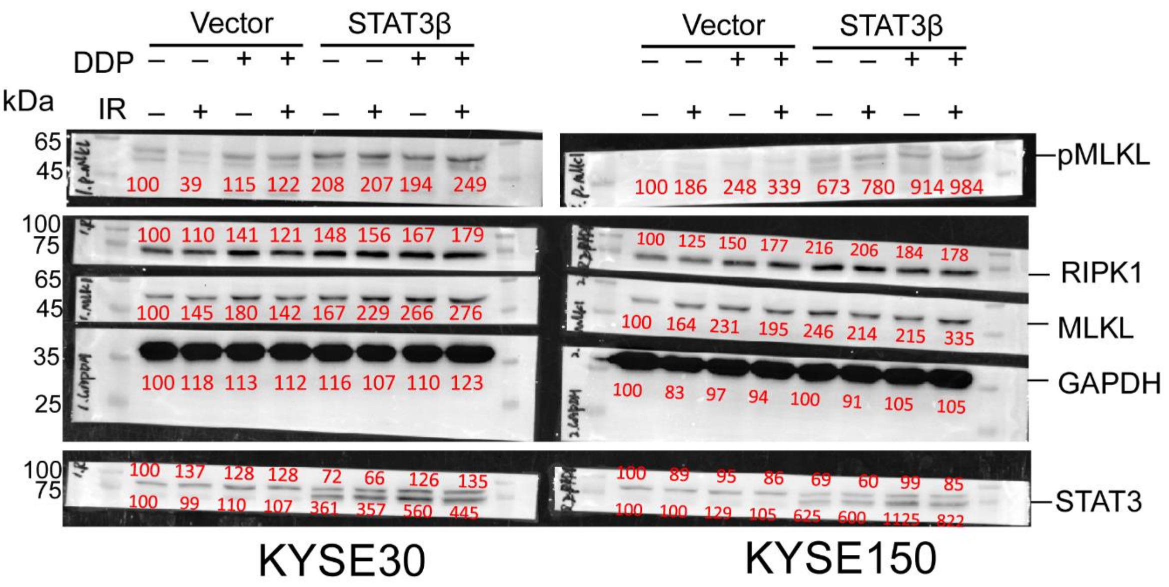

Figure S8. Uncropped Western Blot Images for Figure 5G.

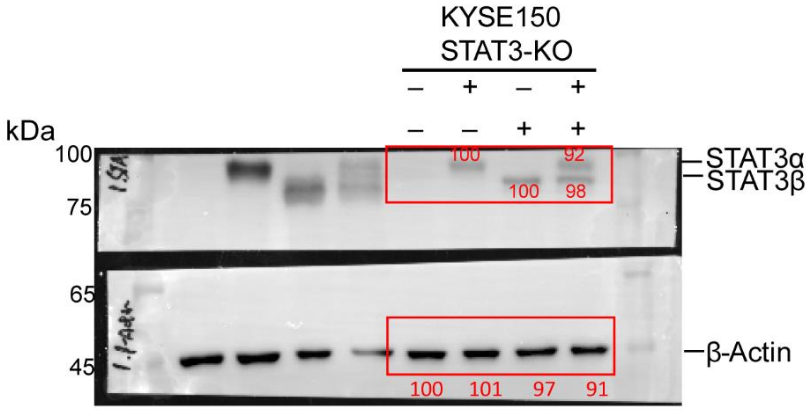

Figure S9. Uncropped Western Blot Images for Figure S1.

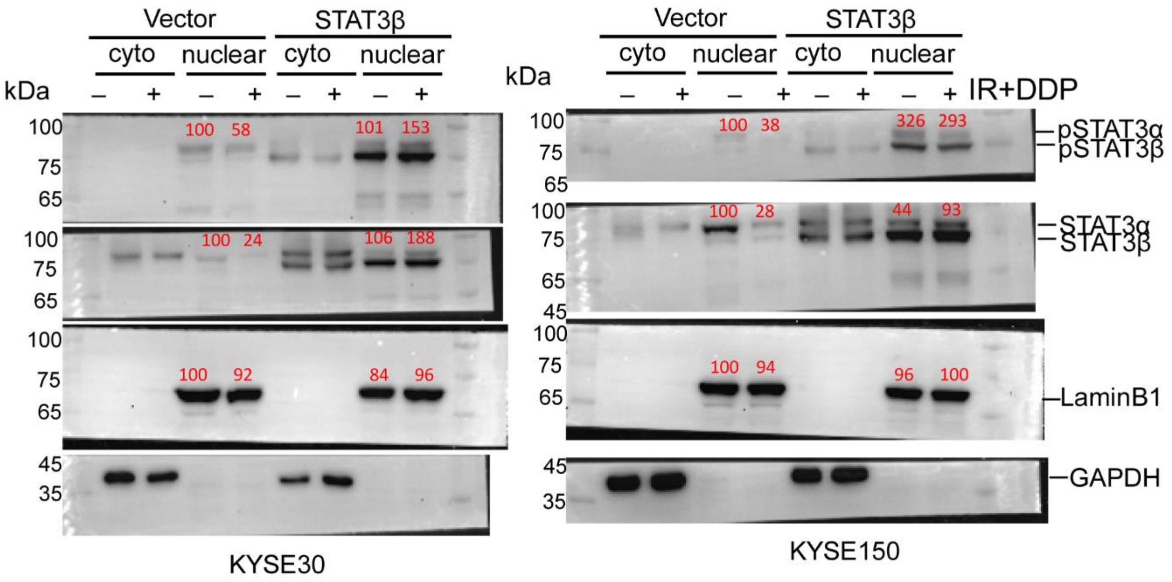

Figure S10. Uncropped Western Blot Images for Figure S3.

Table S1. The correlation between clinical parameters and nuclear STAT3*α* in ESCC patients with concurrent chemoradiotherapy (*n* = 105).

Parameters	Patient Number	Expression of STAT3*α* in Tumor Nucleus		R	*P**
		High[a]	Low[b]		
Gender					
Male	85	25 (29.4%)	60 (70.6%)	0.090	0.359
Female	20	8 (40.0%)	12 (60.0%)		
Age					
≤64.3 years	51	16 (31.4%)	35 (68.6%)	0.001	0.990
>64.3 years	54	17 (31.5%)	37 (68.5%)		
Response					
CR	33	9 (27.3%)	24 (72.7%)	0.061	0.535
PR+SD+PD	72	24 (33.3%)	48 (66.7%)		
Chemoradiotherapy Regimen					
Platinum + IR	64	18 (28.1%)	46 (71.9%)	0.362	0.089
Others	41	15 (36.6%)	26 (63.4%)		
cTNM Classification					
II	19	6 (31.6%)	13 (68.4%)		
III	34	9 (26.5%)	25 (73.5%)	0.044	0.729
IV	52	18 (34.6%)	34 (65.4%)		

[a] high >77.3 scores; [b] Low ≤77.3 scores; CR: complete response; PR: partial response; SD: stable response; PD: progressive disease; IR: ionizing radiation.

Table S2. The correlation between clinical parameters and cytoplasmic STAT3α in ESCC patients with concurrent chemoradiotherapy (n = 105).

Parameters	Patient Number	Expression of STAT3α in Tumor Cytoplasm		R	P*
		High[a]	Low[b]		
		Gender			
Male	85	14 (16.5%)	71 (83.5%)	0.037	0.706
Female	20	4 (25%)	16 (75%)		
		Age			
≤64.3 years	51	8 (15.7%)	43 (84.3%)	0.038	0.700
>64.3 years	54	10 (18.5%)	44 (81.5%)		
		Response			
CR	33	9 (27.3%)	24 (72.7%)	−0.182	0.062
PR+SD+PD	72	9 (12.5%)	63 (87.5%)		
		Chemoradiotherapy Regimen			
Platinum + IR	64	11 (17.2%)	53 (82.8%)	−0.001	0.988
Others	41	7 (17.1%)	34 (82.9%)		
		cTNM Classification			
II	19	3 (15.8%)	16 (84.2%)		
III	34	6 (17.6%)	28 (82.4%)	0.011	0.984
IV	52	9 (17.3%)	43 (82.7%)		

[a] high >126 scores; [b] Low ≤126 scores; CR: complete response; PR: partial response; SD: stable response; PD: progressive disease; IR: ionizing radiation.

Table S3. IC50 of cisplatin from esophageal cancer cells.

Cell lines	IC50 (95%CI) (μM)
KYSE150	44.60 (38.53~52.06)
KYSE450	20.90 (19.58~22.26)
KYSE30	19.25 (16.53~22.38)
KYSE510	12.11 (10.17~14.35)
TE3	5.49 (5.39~5.59)

Table S4. The biological process associated with DEGs affected by high-expressed STAT3β under chemoradiotherapy condition.

Term	−Log(p-value)	Rich factor[a]	Gene symbols[b]
GO: 0051607 [c] defense response to virus	8.486	0.108	GBP1,GBP3,IFI16,IFIT2,IFIT1,IFIT3,IFNB1,IL1B,IL6,CXCL10,IRF1,ISG20,OAS2,TLR3,OASL,FADD,TRIM22,POLR3C,DDX58,IFIT5,DDX60,ZC3HAV1,FAM111A,GPATCH3,RTP4,RSAD2,MLKL,IFNL1,LCN2,IKBKE,IFI44,MR1,IL4R,KRT6A,SERPINE1,S100A9,SLPI,APOL1,NLRP1,GSDMC,NLRC4,RNASE7,PGLYRP3,IL31RA,GSDMA,RESF1,EGR1,IFI35,PSMB8,TRIM21,CXCL8,ZNF502,PAEP,CARD16,CCL2,BNC1,INHBA,PTAFR,LY96,TMEM250,CTSL,ITGA5,SELPLG
GO:0071222 [c] cellular response to lipopolysaccharide	7.950	0.115	ABCA1,CASP1,CXCL1,CXCL2,CXCL3,IL1A,IL1B,IL6,CXCL8,CXCL10,IRAK2,LCN2,LYN,NFKB1,SERPINE1,PTAFR,CCL2,CXCL11,VIM,IL24,LY96,IL36G,PDCD1LG2,CARD16,ADD2,CEACAM1,CD44,CSF1,ITGA5,ITGAM,ITGAX,L1CAM,MMP1,MSN,NBL1,OLR1,PGF,S100A9,SAA1,SDC4,SELPLG,VEGFC,DYSF,FADD,VAV3,VPREB3,ANGPT4,CAMK1D,TMEM102,ABL2,CREB1,DAB2,ESR2,INHBA,NKX3-

			1,P2RY6,SERPINF1,POU4F1,SPP1,ZNF35,NRIP1,TP63,CYP26B1,GRAMD1B,HPGD,SLPI,BMP2,C3,MR1,KRT6A,OAS2,TLR3,IFI44,NLRP1,GSDMC,NLRC4,RNASE7,PGLYRP3,GBP5,LRG1,GSDMA,CCRL2,A2M,C4A,CFH,HPX,IFNB1,PSMB10,C1RL,NRG1,LAMA3,LAMC2,PLAU,PLXNA2,FEZ1,FLRT2,ROBO3,LHX4
GO: 0060337 [c] type I interferon signaling pathway	6.182	0.147	EGR1,IFI35,IFIT2,IFIT1,IFIT3,IFNB1,IRF1,ISG20,OAS2,PSMB8,OASL,FADD,IKBKE,RSAD2
GO:0071396 [c] cellular response to lipid	6.071	0.068	CTSH,CTSL,IFI16,SERPINE1,SERPINE2,PLAU,SERPINF2,FADD,CUZD1,C1RL,NLRC4
GO:0031638 [c] zymogen activation	6.026	0.186	CTSH,CTSL,IFI16,SERPINE1,SERPINE2,PLAU,SERPINF2,FADD,CUZD1,C1RL,NLRC4
GO:1904018 [c] positive regulation of vasculature development	5.215	0.089	CEACAM1,C3,CTSH,EGR1,ENG,FOXC2,IL1A,IL1B,CXCL8,ITGA5,ITGAX,SERPINE1,PDPK1,PGF,SOD2,STAT3,TLR3,VEGFC,ADAM12,ANGPT4,LRG1,AAMP,EDNRA,HPGD,IL6,CXCL10,NKX3-1,PDE2A,SERPINF1,SERPINF2,CCL2,SLC1A1,WARS1,WT1,XDH,DYSF,FZD8,SOCS3,SH2D2A,CALCRL,VAV3,HOXB13,SULF1,ANTXR1,BMP2,CSF1,DAB2,LAMC2,LYN,MMP9,P2RY6,PLAU,PTAFR,FADD,FEZ1,ZNF268,CAMK1D,CEMIP,FERMT3,TMEM102,RNF207,SMIM22,ZNF502
GO:0032612 [c] interleukin-1 production	4.875	0.115	ABCA1,CEACAM1,CASP1,EGR1,MR1,IFI16,IL1B,IL6,SAA1,STAT3,NLRP1,NLRC4,CARD16,GBP5
GO:0043068 [c] positive regulation of programmed cell death	4.380	0.057	BMP2,CASP1,CASP10,CDKN1A,CREB1,CTSH,DUSP6,HPGD,IFIT2,IFNB1,IL6,INHBA,ITGAM,LYN,MCF2,MMP9,NKX3-1,POU4F1,RAPSN,S100A9,CCL2,SOD2,TLR3,XDH,SRPX,TP63,RIPK1,TNFSF10,FADD,SQSTM1,VAV3,ZNF268,NLRP1,CAMK1D,NLRC4,PLEKHF1,RNF122,AIFM2,EGLN3,HCAR2
GO:0045766 [c] positive regulation of angiogenesis	4.374	0.087	C3,CTSH,ENG,FOXC2,IL1A,IL1B,CXCL8,ITGA5,ITGAX,SERPINE1,PDPK1,PGF,STAT3,TLR3,VEGFC,ADAM12,ANGPT4,LRG1
GO:0097527 [c] necroptotic signaling pathway	4.340	0.500	TLR3,RIPK1,FADD,MLKL,IL1B,IL6,SERPINE1,STAT3,DDX58,IL1A,PTAFR,LY96,IKBKE,CSF1,INHBA,MMP9,IL31RA,CEACAM1,CREB1,LYN,POU4F1,MAFB,RFTN1,LCN2,SRPX,IL4R,IL7R,RAG1,NFKBIZ,IFI16
GO:0032611 [c] interleukin-1 beta production	4.166	0.112	ABCA1,CASP1,EGR1,MR1,IFI16,IL1B,IL6,STAT3,NLRP1,NLRC4,CARD16,GBP5
GO:0051092 [c] positive regulation of NF-kappaB transcription factor activity	4.136	0.094	IL1B,IL1RAP,IL6,IRAK2,NFKB1,S100A9,TRIM21,STAT3,TLR3,TRAF1,RIPK1,TRIM22,SLCO3A1,NLRC4,CARD16,CASP1,CASP10,PDPK1,SECTM1,TNFSF10,FADD,SQSTM1,IKBKE,LY96,IFIT5,TNFRSF19,APOL3,ESR2,NKX3-1,DDX58,CAMK1D
GO: 0010720 [d] positive regulation of cell development	10.281	0.082	ABL1,ADRA2C,APBB1,APOE,ASCL1,BCL6,BMP5,BMP7,CRABP2,EDN1,EPHA3,FOXG1,FN1,GATA2,GLI2,FOXA1,HES1,ID2,MDK,MYB,NEDD9,NGFR,NKX6-1,ROR2,RGS2,SCN1B,TP73,CXCR4,FZD1,SEMA7A,KALRN,NEURL1,SLIT2,NTN1,TOX,CUL7,DKK1,PLXND1,COBL,TIAM2,ANKRD1,CDON,PREX1,RNF157,GPC2,SRGAP2C,FOXO6
GO: 0006935[d] chemotaxis	10.184	0.078	APBB1,APOE,BMP5,BMP7,CRABP2,EPHA3,FN1,MDK,NGFR,ROR2,PALM,RGS2,SCN1B,CXCR4,FZD1,SEMA7A,KALRN,NEURL1,SLIT2,NTN1,TOX,CUL7,PLXND1,COBL,DNM3,TIAM2,ANKRD1,ZMYND10,ANLN,ESPN,RNF157,CARMIL2,GPC2,FOXO6
GO: 0040008[d] regulation of growth	7.628	0.0682	APBB1,BCL6,CDKN2C,EPHA7,FGF13,HYAL1,ING1,MAP2,ENPP1,PTPRJ,RGS2,SLIT3,TGFB2,SEMA7A,SLIT2,NTN1,RTN4R,JADE1,PSRC1,DACT3,SEMA3D
GO: 0001558[d] regulation of cell growth	6.349	0.076	ABL1,APBB1,APOE,BCL6,CDKN2C,CRABP2,EDN1,EPHA7,FGF13,FN1,HYAL1,IGFBP3,IGFBP5,ING1,MAP2,NKX6-

			1,ENPP1,PTPRJ,RGS2,SLIT3,TGFB2,CXCR4,SEMA7A,SLIT2,NTN1,RASGRP2,RTN4R,JADE1,PSRC1,RNF157,DACT3,SEMA3D
GO: 0016049 [d] cell growth	6.232	0.071	EFNB3,EPHA7,FBLN1,FGF13,MAP2,NFATC4,NGFR,SEMA7A,SLIT2,NTN1,NGEF,DNM3,RTN4R,SEMA3D
GO: 0050770 [d] regulation of axonogenesis	5.875	0.102	ABL1,APOE,CRABP2,EPHA7,ESR1,FGF13,FN1,LAMB2,MAP2,NKX6-1,SLIT3,CXCR4,SEMA7A,SLIT2,NTN1,SPRY1,COBL,CYFIP2,RTN4R,RNF157,SEMA3D
GO: 0060560 [d] developmental growth involved in morphogenesis	5.452	0.089	ABL1,APOE,CRABP2,EDN1,EPHA7,FGF13,FN1,LAMB2,MAP2,NKX6-1,RGS2,SLIT3,CXCR4,SEMA7A,SLIT2,NTN1,COBL,CYFIP2,RTN4R,RNF157,SEMA3D
GO: 0048588 [d] developmental cell growth	5.423	0.089	EFNB3,EPHA7,FGF13,MAP2,NGFR,SEMA7A,SLIT2,NTN1,RTN4R,SEMA3D

[a] Rich factor, number of differentially expressed genes enriched in GO term/number of total genes in this GO term. The larger rich factor is, the greater the degree of enrichment is. [b] Gene symbols, the threshold of DEGs was ≥1.5-fold change and FDR < 0.05. [c] The genes of biological process enrichment were up-regulated. [d] The genes of biological process enrichment were down-regulated.

Table S5. The KEGG pathway associated with DEGs compared by high-expressed STAT3β under chemoradiotherapy condition.

Term	–Log(*p*-value)	Rich factor[a]	Gene symbols[b]
hsa04668[c] TNF signaling pathway	9.872	0.1739	CASP10, CREB1, CSF1, CXCL1, CXCL2, CXCL3, IFNB1, IL1B, IL6, CXCL10, IRF1, MMP9, NFKB1, CCL2, TRAF1, VEGFC, RIPK1, FADD, SOCS3, MLKL, CASP1, GBP1, GBP3, IFI16, CXCL8, OAS2, IKBKE, NLRP1, NLRC4, ANTXR1, CARD16, GBP5, ANTXR2, LCN2, MMP1, S100A9, C3, ITGAM, C4A, IL1A, ITGA5, LY96, EGR1, SERPINE1, STAT3, PLCD4, CTSL, ATP6V0D1, BDKRB2, LAMA3, LAMC2
ko04621[c] NOD-like receptor signaling pathway	8.990	0.135	CASP1, GBP1, GBP3, CXCL1, CXCL2, CXCL3, IFI16, IFNB1, IL1B, IL6, CXCL8, NFKB1, OAS2, CCL2, RIPK1, FADD, IKBKE, NLRP1, NLRC4, ANTXR1, CARD16, GBP5, ANTXR2
hsa04657[c] IL-17 signaling pathway	6.984	0.158	CXCL1, CXCL2, CXCL3, IL1B, IL6, CXCL8, CXCL10, LCN2, MMP1, MMP9, NFKB1, S100A9, CCL2, FADD, IKBKE
ko04060[c] Cytokine-cytokine receptor interaction	6.373	0.093	BMP2, CSF1, CXCL1, CXCL2, CXCL3, IFNB1, IL1A, IL1B, IL1RAP, IL4R, IL6, IL7R, CXCL8, TNFRSF9, INHBA, CXCL10, CCL2, CXCL11, VEGFC, TNFSF10, OSMR, IL24, IL20RB, TNFRSF19, IFNL1
hsa04610[c] Complement and coagulation cascades	4.977	0.135	A2M, BDKRB2, C3, C4A, CFH, CXCL10, ITGAM, ITGAX, SERPINE1, SERPINB2, PLAU, SERPINF2
ko04620[c] Toll-like receptor signaling pathway	4.974	0.125	IFNB1, IL1B, IL6, CXCL8, CXCL10, NFKB1, CXCL11, SPP1, TLR3, RIPK1, FADD, IKBKE, LY96
hsa04623[c] Cytosolic DNA-sensing pathway	4.815	0.156	CASP1, IFNB1, IL1B, IL6, CXCL10, NFKB1, RIPK1, IKBKE, POLR3C, DDX58
hsa04622[c] RIG-I-like receptor signaling pathway	3.621	0.125	CASP10, IFNB1, CXCL8, CXCL10, NFKB1, RIPK1, FADD, IKBKE, DDX58
hsa04217[c] necroptosis	3.368	0.083	CASP1, GLUD2, IFNB1, IL1A, IL1B, STAT3, STAT6, TLR3, PLA2G4C, RIPK1, TNFSF10, FADD, SQSTM1, MLKL
hsa04390[d] Hippo signaling pathway	4.508	0.104	BMP5, BMP7, BMP8B, CCN2, GLI2, ID2, LLGL2, PPP2R2B, TEAD1, TGFB2, TP73, FZD1, TCF7L1, PARD6B, WTIP, AMOT
hsa04110[d] Cell cycle	4.417	0.113	ABL1, BUB1, CCNA2, CDC20, CDC25C, CDKN2C, GADD45B, PLK1, TGFB2, TTK, WEE1, CCNB2, PTTG1, DBF4
hsa04151[d] PI3K-Akt signaling pathway	2.515	0.061	CHAD, COL4A1, COL4A2, COL6A1, EFNA3, EGF, EPHA2, FGF13, FN1, GNG11, NR4A1, LAMB2, MYB, NGFR, PPP2R2B,

	AKT3, ITGA11, PHLPP2, PKN3, PDGFC, PDGFD, PRKCG, MAP2K6, RAP1GAP, RASGRP2, RASSF5, PARD6B

[a] Rich factor, number of differentially expressed genes enriched in GO term/number of total genes in this GO term. The larger rich factor is, the greater the degree of enrichment is. [b] Gene symbols, the threshold of DEGs was ≥1.5-fold change and FDR < 0.05. [c] The genes of biological process enrichment were up-regulated. [d] The genes of biological process enrichment were down-regulated.

Table S6. The primers sequences used for quantitative RT-PCR.

RIPK1-F	AGATTGGTGGGACGAGTTCAT
RIPK1-R	TGATTGGGTCCAGGTGTTTAT
MLKL-F	CTTGCAGGATTTGAGTTGAG
MLKL-R	GATTTCCCAGAGGACGATTC
IFI16-F	GGAAACTCTGAAGATTGATA
IFI16-R	ATTGTCCTGTCCCCACTACA
IL6-F	GAGGAGACTTGCCTGGTGAA
IL6-R	GGCATTTGTGGTTGGGTCAG
CASP1-F	TTGAAGGACAAACCGAAGGT
CASP1-R	GGAAGAGCAGAAAGCGATAA
IL1B-F	GAATCTCCGACCACCACTAC
IL1B-R	CATAAGCCTCGTTATCCCAT
IL1A-F	GTATGTGACTGCCCAAGATG
IL1A-R	TCCCAGAAGAAGAGGAGGTT
CXCL2-F	CAAACCGAAGTCATAGCCAC
CXCL2-R	GGAACAGCCACCAATAAGCT
MMP9-F	TGCCAGTTTCCATTCATCTTC
MMP9-R	CCCATCACCGTCGAGTCAG

© 2021 by the authors. Licensee MDPI, Basel, Switzerland. This article is an open access article distributed under the terms and conditions of the Creative Commons Attribution (CC BY) license (http://creativecommons.org/licenses/by/4.0/).
